# (*E*)-2-(2-Nitro­ethen­yl)furan

**DOI:** 10.1107/S160053680902861X

**Published:** 2009-07-25

**Authors:** Pedro Valerga, M. Carmen Puerta, Zenaida Rodríguez Negrín, Nilo Castañedo Cancio, Miguel Palma Lovillo

**Affiliations:** a Departamento de Ciencia de los Materiales e Ingeniería Metalúrgica, Facultad de Ciencias, Campus Universitario del Río San Pedro, Puerto Real 11510, Spain; b Centro de Bioactivos Químicos, Universidad Central Marta Abreu de Las, Villas, Cuba; c Departamento de Química Analítica, Facultad de Ciencias, Campus Universitario del Río San Pedro, Puerto Real 11510, Spain

## Abstract

The title compound, C_6_H_5_NO_3_, was synthesized *via* condensation of furfural with nitro­methane in the presence of isobutyl­amine. The compound crystallizes exclusively as the *E* isomer. The angle between the mean planes of the furan ring and the nitro­alkenyl group is 1.3 (2)°.

## Related literature

For general background, see: Wang *et al.* (2009 ▶); An *et al.* (2007 ▶); Rastogi *et al.* (2006 ▶); Rao *et al.* (2005 ▶); Negrín *et al.* (2002 ▶, 2003 ▶); Vallejosa *et al.* (2005 ▶). For related structures, see: Martínez-Bescos *et al.* (2008 ▶); Novoa-de-Armas *et al.* (1997 ▶); Pomes *et al.* (1995 ▶).
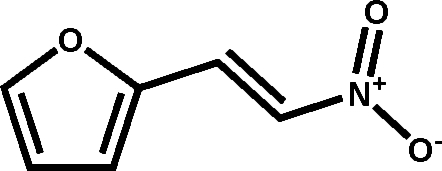


## Experimental

### 

#### Crystal data

C_6_H_5_NO_3_
*M* *_r_* = 139.11
Monoclinic, 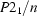

*a* = 9.0374 (18) Å
*b* = 5.2012 (10) Å
*c* = 13.027 (3) Å
β = 97.58 (3)°
*V* = 607.0 (2) Å^3^
*Z* = 4
Mo *K*α radiation
μ = 0.13 mm^−1^
*T* = 100 K
0.47 × 0.17 × 0.14 mm

#### Data collection

Bruker SMART APEX diffractometer
Absorption correction: multi-scan (*SADABS*; Sheldrick, 2004 ▶) *T*_min_ = 0.916, *T*_max_ = 0.980
4852 measured reflections
1387 independent reflections
1317 reflections with *I* > 2σ(*I*)
*R*_int_ = 0.023

#### Refinement

*R*[*F* ^2^ > 2σ(*F* ^2^)] = 0.041
*wR*(*F* ^2^) = 0.107
*S* = 1.06
1387 reflections
91 parameters
H-atom parameters constrained
Δρ_max_ = 0.25 e Å^−3^
Δρ_min_ = −0.28 e Å^−3^

### 

Data collection: *SMART* (Bruker, 2001 ▶); cell refinement: *SAINT* (Bruker, 2001 ▶); data reduction: *SAINT*; program(s) used to solve structure: *SHELXTL* (Sheldrick, 2008 ▶); program(s) used to refine structure: *SHELXTL*; molecular graphics: *ORTEP-3* (Farrugia, 1997 ▶); software used to prepare material for publication: *SHELXTL*.

## Supplementary Material

Crystal structure: contains datablocks global, I. DOI: 10.1107/S160053680902861X/fj2237sup1.cif

Structure factors: contains datablocks I. DOI: 10.1107/S160053680902861X/fj2237Isup2.hkl

Additional supplementary materials:  crystallographic information; 3D view; checkCIF report

## Figures and Tables

**Fig. 1.**
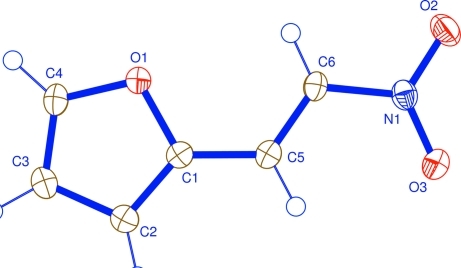
ORTEP representation of the molecular structure of the title compound showing the atom labelling scheme (thermal ellipsoid probability 50%).

**Fig. 2.**
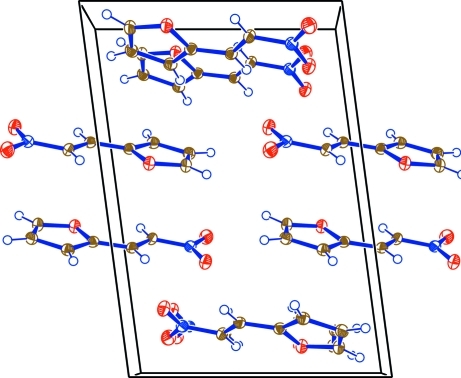
Packing diagram of the title compound.

## supplementary crystallographic information

### Comment

Among the biological properties of (nitro-alkenyl)-furan compounds our interest is focused in their antibacterial and antifungal activities. In spite of the importance of the structure to explain physical and chemical properties, there are not reports on the structures of the more simple compounds in this family. We start with this study a series of structural reports about them. The structure of title compound, showing *trans* or *E* configuration, is shown in Fig. 1. Ring aromaticity is extended to the alkenyl group being C1—C5 bond length, 1.430 (2), significatively shorter than a single C—C bond. Alkenyl *sp*^2^ carbons mantain coplanarity with furan ring as shown by an angle of 1.3 (2)° between ring plane and C5—C6—N1 plane. Crystal packing does not show hydrogen bonds nor N···π intermolecular interactions (Fig. 2).

### Experimental

2-(2-Nitro-ethenyl)-furan, also called G-0, was obtained by a variation of Knoevenagel's method: condensation of an aldehyde with substances containing an active α-hydrogen in the presence of a base (ammonia or amines) as catalyst. The Centro de Bioactivos Químicos (Cuba) has already patented this modified method using furfural, an aromatic compound from acid hydrolisis of sugar cane residuals (straw, sawdust, *etc*.) and nitromethane in the presence of isobutylamine. A yellow crystalline solid was obtained with purity higher than 98%, melting point 74.5°, scarcely soluble in water and very soluble in nitromethane, carbon tetrachloride, petroleum ether and ethanol.

### Refinement

All H atoms were positioned geometrically and treated as riding (C—H = 0.99Å for methylene and C—H = 0.93Å otherwise). *U*_iso_(H) = 1.2 *U*_eq_(C) of the carrier atom.

### Crystal data

**Table:**

C_6_H_5_NO_3_	*F*(000) = 288
*M**_r_* = 139.11	*D*_x_ = 1.522 Mg m^−^^3^
Monoclinic, *P*2_1_/*n*	Mo *K*α radiation, λ = 0.71073 Å
Hall symbol: -P 2yn	Cell parameters from 2396 reflections
*a* = 9.0374 (18) Å	θ = 2.6–27.5°
*b* = 5.2012 (10) Å	µ = 0.13 mm^−^^1^
*c* = 13.027 (3) Å	*T* = 100 K
β = 97.58 (3)°	Prism, yellow
*V* = 607.0 (2) Å^3^	0.47 × 0.17 × 0.14 mm
*Z* = 4	

### Data collection

**Table:**

Bruker SMART APEX diffractometer	1387 independent reflections
Radiation source: fine-focus sealed tube	1317 reflections with *I* > 2σ(*I*)
graphite	*R*_int_ = 0.023
1700 ω scan frames (0.3°, 10)	θ_max_ = 27.5°, θ_min_ = 2.6°
Absorption correction: multi-scan (*SADABS*; Sheldrick, 2004)	*h* = −11→11
*T*_min_ = 0.916, *T*_max_ = 0.980	*k* = −6→6
4852 measured reflections	*l* = −16→12

### Refinement

**Table:**

Refinement on *F*^2^	0 restraints
Least-squares matrix: full	0 constraints
*R*[*F*^2^ > 2σ(*F*^2^)] = 0.041	H-atom parameters constrained
*wR*(*F*^2^) = 0.107	*w* = 1/[σ^2^(*F*_o_^2^) + (0.0569*P*)^2^ + 0.266*P*] where *P* = (*F*_o_^2^ + 2*F*_c_^2^)/3
*S* = 1.06	(Δ/σ)_max_ < 0.001
1387 reflections	Δρ_max_ = 0.25 e Å^−^^3^
91 parameters	Δρ_min_ = −0.28 e Å^−^^3^

### Special details

**Table:**

Experimental. Refinement of F^2^ against unique set of reflections. The weighted *R*-factor *wR* and goodness of fit S are based on F^2^, conventional *R*-factors *R* are based on F, with F set to zero for negative F^2^. The threshold expression of F^2^ > 2sigma(F^2^) is used only for calculating *R*-factors(gt) *etc*. and is not relevant to the choice of reflections for refinement. *R*-factors based on F^2^ are statistically about twice as large as those based on F, and R– factors based on ALL data will be even larger.
Geometry. All e.s.d.'s (except the e.s.d. in the dihedral angle between two l.s. planes) are estimated using the full covariance matrix. The cell e.s.d.'s are taken into account individually in the estimation of e.s.d.'s in distances, angles and torsion angles; correlations between e.s.d.'s in cell parameters are only used when they are defined by crystal symmetry. An approximate (isotropic) treatment of cell e.s.d.'s is used for estimating e.s.d.'s involving l.s. planes.
Refinement. Refinement of *F*^2^ against unique set of reflections. The weighted *R*-factor *wR* and goodness of fit *S* are based on *F*^2^, conventional *R*-factors *R* are based on *F*, with *F* set to zero for negative *F*^2^. The threshold expression of *F*^2^ > σ(*F*^2^) is used only for calculating *R*-factors(gt) *etc*. and is not relevant to the choice of reflections for refinement. *R*-factors based on *F*^2^ are statistically about twice as large as those based on *F*, and *R*- factors based on ALL data will be even larger.

### Fractional atomic coordinates and isotropic or equivalent isotropic displacement parameters (Å^2^)

**Table:**

	*x*	*y*	*z*	*U*_iso_*/*U*_eq_	
O1	0.67495 (10)	0.19614 (17)	0.08110 (7)	0.0206 (2)	
O2	0.15283 (11)	0.49888 (19)	0.10417 (8)	0.0290 (3)	
O3	0.16242 (10)	0.13012 (19)	0.18291 (7)	0.0254 (3)	
N1	0.21943 (12)	0.3007 (2)	0.13567 (8)	0.0205 (3)	
C1	0.59854 (14)	0.0119 (2)	0.12894 (9)	0.0182 (3)	
C2	0.68866 (14)	−0.1941 (2)	0.15531 (9)	0.0202 (3)	
H2	0.6627	−0.3465	0.1890	0.024*	
C3	0.82904 (14)	−0.1370 (3)	0.12270 (10)	0.0221 (3)	
H3	0.9152	−0.2435	0.1300	0.027*	
C4	0.81535 (14)	0.0996 (3)	0.07909 (10)	0.0227 (3)	
H4	0.8930	0.1870	0.0508	0.027*	
C5	0.44755 (13)	0.0618 (2)	0.14455 (9)	0.0185 (3)	
H5	0.3974	−0.0667	0.1787	0.022*	
C6	0.37162 (14)	0.2755 (3)	0.11477 (10)	0.0197 (3)	
H6	0.4172	0.4090	0.0803	0.024*	

### Atomic displacement parameters (Å^2^)

**Table:**

	*U*^11^	*U*^22^	*U*^33^	*U*^12^	*U*^13^	*U*^23^
O1	0.0179 (4)	0.0200 (5)	0.0243 (5)	0.0009 (3)	0.0046 (3)	0.0019 (3)
O2	0.0269 (5)	0.0301 (5)	0.0303 (5)	0.0112 (4)	0.0053 (4)	0.0047 (4)
O3	0.0206 (5)	0.0269 (5)	0.0299 (5)	−0.0014 (4)	0.0074 (4)	0.0019 (4)
N1	0.0189 (5)	0.0244 (6)	0.0182 (5)	0.0026 (4)	0.0023 (4)	−0.0017 (4)
C1	0.0199 (6)	0.0185 (6)	0.0163 (6)	−0.0013 (4)	0.0027 (4)	−0.0017 (4)
C2	0.0221 (6)	0.0199 (6)	0.0186 (6)	0.0006 (5)	0.0019 (5)	−0.0010 (5)
C3	0.0201 (6)	0.0256 (6)	0.0204 (6)	0.0042 (5)	0.0016 (5)	−0.0025 (5)
C4	0.0164 (6)	0.0279 (7)	0.0242 (6)	0.0007 (5)	0.0040 (5)	−0.0011 (5)
C5	0.0185 (6)	0.0212 (6)	0.0159 (6)	−0.0021 (5)	0.0028 (4)	−0.0023 (4)
C6	0.0170 (6)	0.0236 (6)	0.0194 (6)	0.0002 (5)	0.0052 (4)	−0.0013 (5)

### Geometric parameters (Å, °)

**Table:**

O1—C4	1.3680 (15)	C2—H2	0.9500
O1—C1	1.3772 (15)	C3—C4	1.3544 (19)
O2—N1	1.2361 (14)	C3—H3	0.9500
O3—N1	1.2309 (15)	C4—H4	0.9500
N1—C6	1.4428 (16)	C5—C6	1.3366 (18)
C1—C2	1.3623 (17)	C5—H5	0.9500
C1—C5	1.4296 (17)	C6—H6	0.9500
C2—C3	1.4214 (18)		
			
C4—O1—C1	105.97 (10)	C4—C3—H3	126.9
O3—N1—O2	123.33 (11)	C2—C3—H3	126.9
O3—N1—C6	120.08 (11)	C3—C4—O1	111.04 (12)
O2—N1—C6	116.59 (11)	C3—C4—H4	124.5
C2—C1—O1	110.03 (11)	O1—C4—H4	124.5
C2—C1—C5	131.07 (12)	C6—C5—C1	124.94 (12)
O1—C1—C5	118.89 (11)	C6—C5—H5	117.5
C1—C2—C3	106.73 (11)	C1—C5—H5	117.5
C1—C2—H2	126.6	C5—C6—N1	119.11 (12)
C3—C2—H2	126.6	C5—C6—H6	120.4
C4—C3—C2	106.23 (11)	N1—C6—H6	120.4
			
C4—O1—C1—C2	−0.39 (13)	C1—O1—C4—C3	0.54 (14)
C4—O1—C1—C5	178.58 (10)	C2—C1—C5—C6	179.68 (13)
O1—C1—C2—C3	0.12 (14)	O1—C1—C5—C6	0.96 (18)
C5—C1—C2—C3	−178.69 (12)	C1—C5—C6—N1	−179.91 (11)
C1—C2—C3—C4	0.21 (14)	O3—N1—C6—C5	2.03 (17)
C2—C3—C4—O1	−0.47 (14)	O2—N1—C6—C5	−178.15 (11)
